# Physical activity among children with asthma: Cross‐sectional analysis in the UK millennium cohort

**DOI:** 10.1002/ppul.24314

**Published:** 2019-03-18

**Authors:** Katharine C. Pike, Lucy J. Griffiths, Carol Dezateux, Anna Pearce

**Affiliations:** ^1^ Infection, Immunity and Inflammation Academic Programme Great Ormond Street Institute of Child Health, University College London London UK; ^2^ Health Data Research UK, Wales and Northern Ireland Swansea University Medical School Swansea UK; ^3^ Centre for Primary Care and Public Health, Barts and the London School of Medicine and Dentistry Queen Mary University of London London UK; ^4^ MRC/CSO Social and Public Health Sciences Unit University of Glasgow Glasgow UK

**Keywords:** asthma and early wheeze, children, cohort study, epidemiology, physical activity

## Abstract

**Background:**

Although beneficial for health and well‐being, most children do not achieve recommended levels of physical activity. Evidence for children with asthma is mixed, with symptom severity rarely considered. This paper aimed to address this gap.

**Methods:**

We analyzed cross‐sectional associations between physical activity and parent‐reported asthma symptoms and severity for 6497 UK Millennium Cohort Study 7−year‐old participants (3321, [49%] girls). Primary outcomes were daily moderate‐to‐vigorous physical activity (MVPA, minutes) and proportion of children achieving recommended minimum daily levels of 60 minutes of MVPA. Daily steps, sedentary time, and total activity counts per minute (cpm) were recorded, as were parent‐reported asthma symptoms, medications, and recent hospital admissions. Associations were investigated using quantile (continuous outcomes) and Poisson (binary outcomes) regression, adjusting for demographic, socioeconomic, health, and environmental factors.

**Results:**

Neither asthma status nor severity was associated with MVPA; children recently hospitalized for asthma were less likely to achieve recommended daily MVPA (risk ratio [95% confidence interval [CI]]: 0.67 [0.44, 1.03]). Recent wheeze, current asthma, and severe asthma symptoms were associated with fewer sedentary hours (difference in medians [95% CI]: −0.18 [−0.27, −0.08]; −0.14 [−0.24, −0.05]; −0.15, [−0.28, −0.02], respectively) and hospital admission with lower total activity (−48 cpm [−68, −28]).

**Conclusion:**

Children with asthma are as physically active as their asthma‐free counterparts, while those recently hospitalized for asthma are less active. Qualitative studies are needed to understand the perceptions of children and families about physical activity following hospital admission and to inform support and advice needed to maintain active lifestyles for children with asthma.

## 2 INTRODUCTION

The physical and psychological benefits of physical activity are well known.^1^, ^2^ The UK national guidelines recommend young people engage in at least 60 minutes moderate‐to‐vigorous physical activity (MVPA) daily.^3^ Physical activity is likely to be particularly important for children with asthma since exercise training has been shown to reduce exercise‐associated bronchoconstriction^4^ and might also counteract obesity and osteoporosis, which can be associated with inhaled and oral steroid treatments for asthma.^5^, ^6^

Many UK children fail to meet recommended physical activity levels. Physical activity levels vary according to social and demographic factors, most notably gender and ethnicity.^7^ Other factors, including health status, might also influence physical activity. Due to real or perceived limitation, children with chronic conditions might be at increased risk of poor engagement with physical activity; particularly those with conditions, such as asthma, where exercise can exacerbate symptoms.^8^ It remains unclear, however, whether children with asthma are less likely to meet physical activity guidelines than children without asthma, and whether severity of asthma symptoms influences this risk.

A number of studies have considered the relationship between asthma and physical activity. Some have found children with asthma to be less physically active than those without.^9^, ^10^, ^11^ Others, including a recent longitudinal study examining bidirectional associations,^12^ have reported little difference^13^, ^14^ Generally, large population studies including children with asthma of varying severity have found similar levels of physical activity in those with and without asthma.^12^, ^15^, ^16^ Studies reporting lower levels of physical activity in children with asthma compared with their peers without asthma have been small in size,^9^, ^10^, ^11^ relied upon questionnaire‐derived physical activity data,^9^, ^10^, ^11^ or recruited children with asthma severe enough to require follow‐up in a hospital clinic.^9^, ^10^ To our knowledge, no studies have examined objective measures of physical activity or sedentary time in relation to asthma in a large nationally representative sample. Moreover, only a few studies have considered asthma severity^11^, ^14^ or symptom control^17^, ^18^, ^19^ in relation to physical activity.

This study examines associations between parent‐reported asthma and objectively‐measured physical activity and sedentary time in a large, contemporary UK population‐based sample. The association between asthma severity and physical activity is explored using indicators of symptom control, medication use, and history of a recent hospitalization. We hypothesized that children with mild asthma symptoms would be no less active than their peers whereas, for those with more severe asthma, physical activity levels might be lower.

## 3 METHODS

### 3.1 Participants

The Millennium Cohort Study (MCS) recruited 18 818 children born in the United Kingdom between September 2000 and January 2002. Families with eligible children were identified through the Department of Work and Pensions Child Benefit System and selected on the basis of place of residence shortly after the participant child's birth. The sample is clustered at electoral ward level such that families living in the smaller UK countries, in disadvantaged areas, and in England in areas with a high proportion of ethnic minorities are over‐represented.^20^ Main carers for each child were first interviewed when the children were 9 months old and follow‐up data were collected at successive surveys carried out throughout childhood. For 98% of children, the main‐carer respondent was the child's mother.

Of the original cohort, 14 043 (70%) participated in the fourth survey, at 7 to 8 years of age. The main carer for each child participated in a computer‐assisted home‐based interview.

### 3.2 Physical activity outcomes

At the fourth survey, children were invited to participate in the accelerometry study. Those whose parents consented were posted an accelerometer. Of the 13 219 children who agreed to participate in the accelerometer study, 7004, 6675, 6326, 5910, 5153, 4002, and 2244 had ≥1, ≥2, ≥3, ≥4, ≥5, ≥6, and ≥7 reliable days of data lasting at least 10 hr/day.^21^ This analysis was restricted to 6497 singleton children with at least 2 days with 10 or more hours of data each. Activity was measured using the Actigraph GT1M Uni‐axial Accelerometer (Actigraph, Pensacola, FL), initialized using ActiLife Lifestyle Monitoring System software version 3.2.11 (Actigraph) and programmed to use a 15‐second sampling epoch to record activity counts. Children were asked to wear their accelerometer on their right hip during all waking hours from the morning after they received it for 7 consecutive days (except when in contact with water). Data were collected between May 2008 and August 2009 and downloaded using Actigraph software V.3.8.3 (Actigraph). Nonwear time, defined as consecutive zero activity counts greater than or equal to 20 minutes, was excluded from analysis as extreme count values were greater than or equal to 11 715 counts per minute (cpm).^22^

Threshold values for the accelerometer cpm were defined as less than 100 for sedentary time, and less than or equal to 2240, less than or equal to 3840, and greater than or equal to 3841 for light, moderate, and vigorous physical activity, respectively.^23^ Since the total valid recording time was not constant across days, total values for counts were standardized with reference to a standard day of equal duration for all children.^24^ The primary outcome was mean daily minutes spent in MVPA. The proportion of children meeting the national recommendation of greater than or equal to 60 minutes MVPA daily was also calculated. Secondary outcomes were mean daily hours spent sedentary, mean daily steps, and total activity level (cpm).

### 3.3 Exposure variables: asthma and asthma severity

Exposure variables were based upon parental response to questions from the International Study of Asthma and Allergy in Childhood (ISAAC) core questionnaire for asthma age 6 to 7 years^25^ and additional questions about medication use and hospital admission. All were measured at the 7‐year survey.

### 3.4 Asthma

“Ever asthma” and “recent wheeze” status were assigned according to the children's main‐carers’ responses to respectively “has your child ever had asthma?” and “has your child had wheezing or whistling in the chest in the last 12 months.” “Current asthma” status was a derived variable requiring carers to affirm their child as ever having asthma, and wheezing in the last 12 months or using asthma medications (based on reported use of “any medications on a regular basis (including inhalers) that were prescribed by a doctor or hospital?” and including relievers or preventers identified via British National Formulary codes [Appendix S1]).

### 3.5 Asthma severity

Children were categorized using the ISAAC definition of severe asthma and two further derived variables based upon reported asthma treatment or recent hospital admission for an asthma attack. To determine severity according to the ISAAC criteria carers were asked: “how many attacks of wheezing has your child had in the last 12 months?” (none, 1‐3, 4‐12, or >12). They were then asked, “in the last 12 months, how often on average has your child's sleep had been disturbed by wheezing?” (never, <1 night/week, or ≥1 night/week), and “in the last 12 months, has wheezing been bad enough to limit your child's speech to one or two words at a time between breaths?” (yes or no). Those reporting greater than or equal to four attacks of wheeze per year, or greater than or equal to one night/week of sleep disturbance due to wheeze, or wheeze affecting speech were classified as having severe asthma.^26^ Those reporting prescription of any inhaled corticosteroid were compared with those who did not. Finally, carers reported whether the child had been admitted to hospital since last interviewed at age 5 years and reported the reason for the most serious admission. This information was used to categorize children as having experienced a recent hospital admission due to “asthma or wheezing” (compared to those not admitted to hospital or for whom their most serious admission was for a condition other than asthma).

### 3.6 Confounding factors

A number of potential common causes of asthma and physical activity were adjusted for a priori, as outlined in the directed acyclic graph (DAG)^27^ (shown in Figure S1). Most were based on information collected from the main carer when the child was aged 7 years: child's sex, ethnicity, socioeconomic status (main carer's last employment or partner's if higher), number of siblings living in the household, country of residence at interview (England, Scotland, Northern Ireland, or Wales), and household tobacco smoke exposure. Body mass index (BMI) was calculated for each child at age 7 from measured weights and heights, which were converted to age‐ and sex‐adjusted *z* scores using the UK 1990 growth reference.^28^

### 3.7 Statistical analysis

The association between asthma status and physical activity was investigated before and after adjustment for confounding in multivariable models. As physical activity outcomes were positively skewed quantile regression was used to compare median differences between children categorized according to asthma status and asthma severity for the following outcomes, all measured daily: minutes of MVPA, total activity (cpm), hours of sedentary time, and total steps. Risk ratios (RR) (and 95% confidence intervals [CI]) were estimated in multivariable Poisson regression, to compare the proportion of children with and without asthma who met the recommended physical activity level of 60 minutes of MVPA daily. Survey weights were used to adjust for nonresponse and study sampling design.^20^, ^24^ A sensitivity analysis was performed excluding BMI since this might have a bidirectional association with physical activity level.

## 4 RESULTS

Of the 6497 children with valid accelerometer data, 6479 (99.6%) also had data from one or more of the asthma questions; of these, 979 (16.0%) reported ever receiving an asthma diagnosis, 743 (12.1%) reported wheeze within the last 12 months, and 512 (8.4%) reported current asthma. Severe asthma symptoms according to ISAAC criteria were reported by 323 (5.3%) children, 155 (2.5%) children reported being prescribed an inhaled corticosteroid, and 51 (0.7%) reported a hospital admission for asthma since last interviewed at 5 years. Median (IQR) values for activity outcomes were 594 cpm (504 to 697) daily total activity, 6.4 (5.8‐7.1) hours spent sedentary, 60.5 (46.9‐76.1) minutes spent in MVPA, and 10131 (8653‐11794) steps daily. Including children with and without asthma, the proportion meeting recommended activity levels of 60 minutes MVPA daily was 50.9 (95% CI, 49.1‐52.6). Neither asthma prevalence, nor sociodemographic, nor other explanatory variables differed appreciably between the complete data set of singleton children interviewed at 7 years and the smaller subset of children with activity data (Table 1).

**Table 1:** Comparison of asthma status and sociodemographic characteristics between the whole cohort and the subset of singleton children with accelerometer data

Variables	All singleton children who participated in the age 7 survey			Subset singletons with physical activity data		
	Percentages weighted according to survey weights			Percentages weighted according to accelerometer study weights		
	Male	Female	Total	Male	Female	Total
N (%)	6950 (51.4)	6731 (48.6)	13681	3176 (51.1)	3321 (48.9)	6497
Asthma ever						
No	5572 (80.8)	5761 (86.6)	11333 (83.6)	2597 (81.4)	2903 (86.7)	5500 (84.0)
Yes	1131 (19.2)	912 (13.4)	2243 (16.4)	570 (18.6)	409 (13.3)	979 (16.0)
Recent wheeze						
No	5927 (86.0)	6019 (90.3)	11946 (88.1)	2736 (85.9)	3009 (90.0)	5745 (87.9)
Yes	989 (14.0)	667 (9.7)	1656 (11.9)	435 (14.1)	308 (10.0)	743 (12.1)
Current asthma						
No	6181 (89.84)	6208 (93.1)	12389 (91.4)	2862 (90.0)	3103 (93.26)	5965 (91.6)
Yes	719 (10.2)	464 (6.9)	1183 (8.6)	304 (10.0)	208 (6.8)	512 (8.4)
Severe asthma symptoms (ISAAC)						
No	6446 (93.5)	6382 (95.7)	12828 (94.6)	2896 (93.9)	3179 (95.5)	6165 (94.7)
Yes	470 (6.5)	304 (4.3)	774 (5.4)	185 (6.1)	138 (4.5)	323 (5.3)
Prescribed inhaled corticosteroids						
No	6742 (97.4)	6550 (98.0)	13292 (97.7)	3088 (97.4)	3241 (97.7)	6329 (97.5)
Yes	174 (2.6)	129 (2.0)	303 (2.3)	2 (2.6)	73 (2.3)	155 (2.5)
Asthma admission since age 5 y						
No	6832 (98.9)	6642 (99.4)	13474 (99.1)	3134 (99.1)	3301 (99.5)	6435 (99.3)
Yes	84 (1.1)	44 (0.6)	128 (0.9)	35 (0.9)	16 (0.5)	51 (0.7)
Country						
England	4451 (63.9)	4335 (64.6)	8786 (64.2)	2064 (64.7)	2140 (65.2)	4204 (64.9)
Wales	1015 (14.8)	930 (13.6)	1945 (14.2)	450 (14.6)	448 (13.1)	898 (13.8)
Scotland	809 (11.3)	790 (12.0)	1599 (11.6)	363 (11.1)	398 (11.9)	761 (11.5)
Northern Ireland	675 (10.0)	676 (9.8)	1351 (9.9)	299 (9.6)	335 (9.8)	634 (9.7)
Socioeconomic classification (highest of main carer and partner)						
Managerial and professional	3112 (46.2)	2978 (45.9)	6090 (46.1)	1743 (47.4)	1721 (45.0)	3464 (46.2)
Intermediate	937 (13.5)	854 (13.4)	1791 (13.5)	418 (13.7)	430 (13.1)	848 (13.4)
Self‐used	650 (10.0	661 (10.1)	1311 (10.1)	268 (9.7)	308 (10.0)	576 (9.8)
Low supervisory and technical	509 (7.7)	497 (7.6)	1006 (7.7)	205 (7.4)	237 (8.6)	442 (8.0)
Semi‐routine and routine	1487 (22.5)	1489 (22.9)	2976 (22.7)	480 (21.7)	560 (23.2)	1040 (22.5)
Ethnicity						
White	5830 (85.6)	5611 (85.3)	11441 (85.4)	2795 (85.0)	2916 (85.4)	5711 (85.2)
Mixed	178 (3.1)	194 (3.4)	372 (3.2)	85 (3.5)	83 (3.0)	168 (3.2)
Indian	179 (2.1)	162 (1.8)	341 (1.9)	61 (1.8)	78 (2.2)	139 (2.0)
Pakistani/Bangladeshi	421 (4.5)	462 (5.0)	883 (4.7)	123 (5.4)	124 (4.9)	247 (5.1)
Black	243 (3.5)	209 (3.1)	452 (3.3)	76 (3.2)	76 (2.9)	152 (3.0)
Other	99 (1.3)	92 (1.5)	191 (1.4)	36 (1.2)	44 (1.7)	80 (1.4)
Number of children in the household						
Other children in the household	851 (12.5)	808 (12.4)	1659 (12.4)	324 (11.3)	355 (12.0)	679 (11.6)
Only child	6099 (87.5)	5923 (87.6)	12022 (87.6)	2852 (88.7)	2966 (88.0)	5818 (88.4)
BMI (z score)						
Mean (95% CI)	0.40 (0.37, 0.44)	0.32 (0.29, 0.36)	0.36 (0.34, 0.39)	0.39 (0.34, 0.44)	0.30 (0.25, 0.35)	0.35 (0.31, 0.38)
BMI, kg/m^2^						
Median (IQR)	16.1 (16.0, 16.2)	16.2 (16.1, 16.2)	16.1 (16.1, 16.2)	16.0 (15.9, 16.1)	16.1 (16.0, 16.2)	16.1 (16.0, 16.2)
Household spoking exposure						
No	6068 (87.1)	5769 (85.7)	11837 (86.4)	2852 (87.4)	2935 (85.6)	5787 (86.5)
Yes	847 (12.9)	912 (14.3)	1759 (13.6)	320 (12.6)	381 (14.4)	701 (13.5)

Abbreviations: BMI, body mass index; CI, confidence interval; IQR, interquartile range.

Missing data (main dataset, data set, physical activity subset): asthma ever 105, 18; recent wheeze 79, 9; current asthma 109, 20; severe asthma symptoms (ISAAC) 79, 9; hospital admission for asthma since age 5 years 79, 11; prescribed inhaled corticosteroids 86, 13; socioeconomic status 507, 127; body mass index 221; 21 household smoking exposure 85, 9.

### 4.1 Physical activity levels according to asthma status

Neither 'has ever received a diagnosis of asthma', nor 'current asthma' was associated with MVPA (Table 2). Children reported to have experienced wheeze within the last 12 months recorded more daily minutes of MVPA (difference in medians [95% CI]: 3.8 [1.0, 6.5]) but this association was attenuated and did not remain significant after adjustment (0.5 [−1.6‐2.6]) (Table 2). The proportion of children attaining daily recommended physical activity levels was low regardless of asthma status (Table 3). Of children reported to have ever received a diagnosis of asthma, 53.4% (95% CI: 49.4, 57.4) met recommendations compared with 50.3% (48.6‐52.0) of children reported never to have been diagnosed with asthma. Similar patterns were observed for reported recent wheeze and current asthma.

**Table 2:** Difference in median values for daily number of minutes spent in moderate/vigorous activity according to asthma and wheeze status

Asthma status/severity	Unadjusted			Adjusted^a^		
	Difference in medians (95% CI)	*P* value	*n*	Difference in medians (95% CI)	*P* value	*n*
Asthma ever	1.3 (−0.8, 3.5)	0.232	6479	−1.4 (−3.6, 0.9)	0.230	6329
Recent wheeze	3.8 (1.0, 6.5)	0.007	6488	0.5 (−1.6, 2.6)	0.646	6338
Current asthma	2.3 (−0.7, 5.4)	0.137	6477	−0.5 (−3.4, 2.3)	0.715	6327
Severe asthma symptoms (ISAAC)	3.5 (0.4, 7.3)	0.078	6488	0.0 (−4.4, 4.5)	0.987	6338
Prescribed inhaled corticosteroids	0.2 (6.0, 6.5)	0.942	6484	−2.8 (−.7, 2.1)	0.267	6334
Asthma admission since age 5 y	‐7.1 (–13.6, ‐0.5)	0.034	6486	−5.1 (−13.9, 3.7)	0.258	6336

Abbreviation: CI, confidence interval.

^a^ Weighted for nonresponse and study sampling design and adjustment made for body mass index, sex, socioeconomic status, ethnicity, the presence of other children in the household, country of residence, and household smoking exposure.

**Table 3:** Children meeting daily activity guidelines for moderate‐to‐vigorous activity: weighted proportions and adjusted risk ratios according to asthma status and severity

Asthma status/severity	Weighted proportion % (95% CI)^a^		RR (95% CI)^a^, ^b^	*P* value	*n*
	Yes	No			
Asthma ever	53.4 (49.4, 57.4)	50.3 (48.6, 52.0)	0.99 (0.8911.07)	0.730	6329
Recent wheeze	56.9 (52.44 61.3)	50.0 (48.3, 51.6)	1.06 (0.97, 1.15)	0.231	6338
Current asthma	54.9 (49.4, 60.3)	50.4 (48.8, 52.1)	1.02 (0.92, 1.13)	0.721	6327
Severe asthma symptoms (ISAAC)	57.2 (50.6, 63.8)	50.5 (48.9, 52.1)	1.08 (0.95, 1.22)	0.226	6338
Prescribed inhaled corticosteroids	50.1 (40.5, 59.6)	50.8 (49.2, 52.4)	0.93 (0.77, 1.12)	0.443	6334
Asthma admission since age 5 y	36.0 (19.8, 52.2)	50.9 (49.4, 52.5)	0.67 (0.44, 1.03)	0.069	6336

Abbreviations: CI, confidence interval.

^a^ Weighted for nonresponse and noncompliance as well as study sampling design.

^b^ Adjusted for body mass index, sex, socioeconomic status, ethnicity, the presence of other children in the household, country of residence, and exposure to household tobacco smoke.

Children reported to have experienced recent wheeze recorded greater total activity (cpm) and total daily steps than those without recently reported wheeze (difference in medians [95% CI]: 20 [2, 38] and 329 [39‐618], respectively), but these associations were attenuated and neither remained significant after adjustment (Table S1). The inclusion of socioeconomic status and gender in the adjusted model accounted for most of this attenuation. Children reported to have had recent wheeze also spent fewer daily hours sedentary (difference in medians [95% CI]: −0.21 [−0.30, −0.11]) with little change after adjustment (−0.18 [−0.27 , −0.08]) (Table S1). Reports of ever asthma and current asthma were also associated with less daily sedentary time; however, both associations attenuated after adjustment with small associations with current asthma remaining (difference in medians [95% CI]: −0.14 [−0.24, −0.05]).

### 4.2 Physical activity levels according to asthma severity

Neither 'severe asthma symptoms' (according to ISAAC criteria) nor 'prescription of inhaled corticosteroids' was associated with daily MVPA. Children with a previous hospital admission for asthma reported fewer daily minutes of MVPA (difference in medians [95% CI]: −7.1 [−13.6, −0.5]) but this association was partly attenuated and no longer significant after adjustment (−5.1 [−13.9, 3.7]) (Table 2). Proportions meeting national guidelines did not vary by severe asthma symptoms or prescribed inhaled corticosteroids (Table 3). Of those reporting an asthma admission since the previous interview the proportion meeting national guidelines was 36.0% (95% CI, 19.8, 52.2) compared with 50.9% (49.4‐52.5) among those who did not report an admission (RR [95% CI]: 0.67 [0.44, 1.03]) (Table 3).

Children with carer‐reported hospital admission due to asthma since last interviewed were less active than those without such an admission (difference in medians [95% CI]: −48 cpm [−68, −28]). (Table S2). In contrast, both children prescribed inhaled corticosteroids and those reported to have severe asthma symptoms spent fewer hours sedentary than their comparison groups. However both associations were attenuated after adjustment and only a small association with severe asthma symptoms remained (−0.15 [−0.28, −0.02]).

Direction and size of main effects were unaffected by excluding BMI from the multivariable model.

## 5 DISCUSSION

This study is the first to compare physical activity levels among children with and without asthma in a large prospective, representative UK‐wide cohort, using objectively‐measured activity data from 6497 7 to 8‐year‐old children. Importantly, in contrast to cohorts drawn from clinical samples, the MCS sampling and study design did not select children with severe or poorly controlled asthma. Approximately half of all children who had ever received an asthma diagnosis did not meet recommended levels of 60 minutes daily MVPA; this is similar to the proportion for all children reported previously.^7^ This proportion did not vary according to any of the asthma measures or by asthma severity, with the exception of children who hospitalized in the preceding 2 years for asthma. These children were less likely to meet UK guidelines and engaged in less total activity than those not reporting an admission. Sedentary time was less in children reporting recent wheeze, current asthma, and severe asthma symptoms.

Our main finding that physical activity differs little between children with and without asthma differs from some previous studies which have reported reduced physical activity in children with asthma. These studies have for the most part recruited from pediatric outpatient clinics rather than from nationally representative population samples. Most used questionnaire‐derived activity data and included small numbers of children, potentially with relatively severe or uncontrolled symptoms.^9^, ^10^ Of the few large population studies to have considered physical activity in children with asthma, many have relied upon questionnaire‐derived activity data and unvalidated parental or self‐reports of asthma status. These studies also found little evidence of reduced physical activity in association with asthma.^12^, ^15^, ^16^ In contrast to our findings, some population studies have found positive associations between asthma and sedentary time, in particular with computer use or television viewing.^15^, ^16^, ^29^ Our findings are consistent, however, with a recent meta‐analysis of studies objectively measuring physical activity and a report from the Avon Longitudinal Study of Parents and Children (ALSPAC); both found no evidence that physical activity levels differed between children with and without asthma.^30^, ^31^

In our study, the only exposure associated with lower physical activity was parent‐reported hospital admission for asthma. Children previously admitted to hospital for asthma engaged in less total activity than those without a history of such an admission and were also less likely to meet national physical activity guidelines. Reduced physical activity was not associated with severity based on epidemiological criteria used in ISAAC or upon regular use of prescribed inhaled corticosteroids. Since a previous asthma attack is the strongest predictor of future attacks,^32^ children who had experienced a hospital admission might be those most at risk and potentially fearful of future attacks. Moreover, where attacks occur due to poor asthma control, interval symptoms might restrict physical activity. This group included fewer children than the other indicators of severity. While this might serve to identify children with the most severe asthma, the precision of outcome estimates for this group was low.

Generally, asthma severity is quantified according to the level of treatment needed to achieve symptom control. However associations between disease severity, medication prescription, and clinical symptoms are complex since loss of symptom control can occur, because of under‐prescribing, poor inhaler technique, nonadherence to medication, or severe or treatment‐resistant disease. Studies which have compared physical activity in children with severe or poorly controlled asthma to that in those with the less severe or well‐controlled disease have reported conflicting results; some have found reduced physical activity in association with more severe disease,^11^ or poorer control,^18^ while others have found no association.^14^, ^17^ It is possible that previous hospital admission is a stronger influence upon physical activity than measures of medication or symptoms because it is a strong predictor of future attacks and this is something children with asthma (and their parents) wish to avoid.

While children reporting recent wheeze, current asthma, and severe asthma symptoms were no more or less active than their peers, they did engage in less sedentary time. However, these differences were small compared to the overall sedentary time and are of uncertain clinical significance. It is possible that given the promotion of physical activity within asthma management guidelines^33^ the associations seen between asthma status and less time spent sedentary might arise because parents of children with asthma might encourage light activity over sedentary time.

### 5.1 Strengths and limitations

The findings from this study do not provide any evidence that children with asthma are less physically active than those without asthma, with the exception of the small proportion previously admitted to hospital for asthma who tended to be less active overall. Some studies have suggested a causal role for sedentary behavior in asthma pathogenesis,^29^, ^34^ while others have suggested that the likelihood of receiving asthma or wheeze diagnosis might be greater for physically active children.^15^ Due to the cross‐sectional nature of the MCS data we have analyzed, it is not possible to determine the direction of this relationship and this was not the purpose of our study. In this study we measured physical activity using a type of accelerometer that has been validated against heart rate monitoring, calorimetry and energy expenditure measured using doubly labeled water.^35^ Specific activity count thresholds were calibrated for the device used and the participant age group.^23^ Potential limitations of the study were an underestimation of activity due to the need to remove the accelerometers during water‐based or contact sports and risk of over‐interpretation because asthma symptoms were not recorded concurrently with physical activity. Moreover, relying upon self‐reported asthma status introduces the possibility for misclassification. Prevalence estimates reflect the methods used to estimate asthma prevalence as discussed by Punekar and Sheikh^36^ and as highlighted in our previous study comparing the prevalence of parent‐reported with doctor‐diagnosed asthma. This showed moderate‐to substantial agreement between parentally‐reported and doctor‐diagnosed asthma at this age in this cohort.^37^ Nevertheless, the results of this study should be interpreted as a reflection of activity levels among children with parent‐reported asthma.

### 5.2 Clinical implications

While a comparable proportion of children with and without asthma failed to achieve the recommended physical activity levels, those who had experienced recent hospitalization due to asthma were less likely to be moderately or vigorously active for 60 minutes or more each day. As shown previously,^7^ half of UK children fail to meet recommended physical activity levels but the reasons for poor engagement might differ according to health status. Addressing fears surrounding exercise‐induced asthma might be necessary to engage children with asthma with interventions designed to increase physical activity.^38^ Physical training has been demonstrated not to worsen airway inflammation in children with asthma,^39^ exercise‐induced bronchoconstriction is seen more often in those with poorly than well‐controlled asthma,^40^ and greater physical activity has been shown to follow improved asthma control.^19^ Therefore, providing children with asthma and their parents, teachers, and healthcare professionals, with information about the benefits of gaining symptom control, and supporting them in achieving this would appear to be important components of any intervention specifically targeting children with asthma. Positively, children with asthma and severe asthma were less sedentary, possibly indicating that families may be taking steps to replace sedentary behaviors with light physical activity. They thus may be receptive to further increases in physical activity, where health professionals have identified greater physical activity as being appropriate and of benefit to that individual.

In conclusion, we found no evidence to suggest that children in the general population with asthma—as reported by their parent—are less physically active than those without asthma and that they are slightly less sedentary. There is some suggestion from our findings that the very small percentage of children with asthma severe enough to require hospital admission are less active and thus less likely to meet national guidelines to achieve a minimum of an hour of moderate and vigorous activity daily. When designing interventions to increase physical activity among children with asthma, children with admission to hospital for an asthma attack might be at greatest risk of poor engagement. Further work is required to identify the real or perceived barriers to engagement in this and other at‐risk groups. Lower sedentary behavior among children with asthma indicates that families may be receptive to increasing physical activity, with the right information, guidance, and support from health professionals.

## AUTHOR CONTRIBUTIONS

CD and KP are coinvestigators and members of the Asthma UK Centre for Applied Research (grant reference AUK‐AC‐2018‐01). LG and CD are supported by Health Data Research UK, which is funded by the UK Medical Research Council, Engineering and Physical Sciences Research Council, Economic and Social Research Council, National Institute for Health Research (England), Chief Scientist Office of the Scottish Government Health and Social Care Directorates, Health and Social Care Research, and Development Division (Welsh Government), Public Health Agency (Northern Ireland), British Heart Foundation, and Wellcome. AP is funded by a Wellcome Trust University Award (205412/Z/16/Z). She also receives support from the Medical Research Council (MC_UU_12017/13) and the Scottish Government Chief Scientist Office (SPHSU13).

## DATA AVAILABILITY

All MCS data used in this analysis are available from UK Data Service, University of Essex and University of Manchester: http://doi.org/10.5255/UKDA‐SN‐8172‐2, http://doi.org/10.5255/UKDA‐SN‐6411‐7, and http://doi.org/10.5255/UKDA‐SN‐72

## Supporting information

Supporting information

Supporting information

Supporting information

Supporting information
