# Supplementary material for: Physical activity among children with asthma: Cross‐sectional analysis in the UK millennium cohort
Source: Pediatr Pulmonol. 2019 Mar 18;54(7):962–9. doi: 10.1002/ppul.24314 (PMC6617805; doi:10.1002/ppul.24314)
Supplement: Supplementary file 1 — Supporting information [file PPUL-54-962-s001.docx]

**The relationship between asthma and physical activity during childhood: a cross-sectional analysis**

Katharine C Pike^1^, Lucy J Griffiths^2^, Carol Dezateux^3^, Anna Pearce^4^

^1^Infection, Immunity and Inflammation Academic Programme, Great Ormond Street Institute of Child Health, University College London, London, UK; ^2^Health Data Research UK, Wales and Northern Ireland, Swansea University Medical School, Swansea, UK; ^3^Centre for Primary Care and Public Health, Barts and the London School of Medicine and Dentistry, Queen Mary University of London, London, UK; ^4^MRC/CSO Social and Public Health Sciences Unit, University of Glasgow, Glasgow, UK.

Corresponding author: Dr Katharine Pike

Infection, Immunity and Inflammation Academic Programme, Great Ormond Street Institute of Child Health, 30 Guilford Street, London, WC1N 1EH, UK

Phone: +44 (0)20 7905 2376

Keywords: asthma, physical activity, wheeze, cohort study, children

Appendix S1 - BNF medication codes

Bronchodilators: 030101 Adrenoceptor agonists, 030102 Antimuscarinc bronchodilators

Inhaled steroid preventer: 030200 Corticosteroids

Other asthma medication: 030302 Leukotriene receptor antagonists, 030103 Theophylline
