# Supplementary material for: Physical activity among children with asthma: Cross‐sectional analysis in the UK millennium cohort
Source: Pediatr Pulmonol. 2019 Mar 18;54(7):962–9. doi: 10.1002/ppul.24314 (PMC6617805; doi:10.1002/ppul.24314)
Supplement: Supplementary file 2 — Supporting information [file PPUL-54-962-s002.docx]

Table S1 Secondary activity outcomes according to asthma and wheeze status

| Asthma status / severity | Unadjusted | | | Adjusted^a^ | | |
| --- | --- | --- | --- | --- | --- | --- |
|  | Difference in Medians (95% CI) | p-value | n | Difference in Medians (95% CI) | p-value | n |
|  | Total activity (counts per minute) | | | | | |
| Asthma ever | 11 (-4, 25) | 0.143 | 6479 | -7 (-23, 10) | 0.406 | 6329 |
| Recent wheeze | 20 (2, 38) | 0.033 | 6488 | 0 (-19, 18) | 0.982 | 6338 |
| Current asthma | 8 (-11, 27) | 0.398 | 6477 | -9 (-30, 11) | 0.357 | 6327 |
|  | Daily number of hours sedentary | | | | | |
| Asthma ever | -0.13 (-0.22, -0.03) | 0.008 | 6479 | -0.06 (-0.14, 0.02) | 0.140 | 6329 |
| Recent wheeze | -0.21 (-0.30, -0.11) | <0.001 | 6488 | -0.18 (-0.27, -0.08) | <0.001 | 6338 |
| Current asthma | -0.18 (-0.29, -0.07) | 0.001 | 6477 | -0.14 (-0.24, -0.05) | 0.002 | 6327 |
|  | Daily total steps | | | | | |
| Asthma ever | 235 (-42, 512) | 0.096 | 6479 | 71 (-194, 334) | 0.595 | 6329 |
| Recent wheeze | 329 (39, 618) | 0.026 | 6488 | 185 (-69, 439) | 0.154 | 6338 |
| Current asthma | 236 (-153, 625) | 0.234 | 6477 | 81 (-213, 376) | 0.589 | 6327 |

^a^Adjustment made for BMI, sex, socioeconomic status, ethnicity, presence of other children in the household, country of residence and household smoking exposure.
