# Supplementary material for: Physical activity among children with asthma: Cross‐sectional analysis in the UK millennium cohort
Source: Pediatr Pulmonol. 2019 Mar 18;54(7):962–9. doi: 10.1002/ppul.24314 (PMC6617805; doi:10.1002/ppul.24314)
Supplement: Supplementary file 3 — Supporting information [file PPUL-54-962-s003.docx]

Table S2 Secondary activity outcomes according to alternative measures of asthma severity

| Asthma status / severity | Unadjusted | | | Adjusted^a^ | | |
| --- | --- | --- | --- | --- | --- | --- |
|  | Difference in Medians (95% CI) | p-value | n | Difference in Medians (95% CI) | p-value | n |
|  | Total activity (counts per minute) | | | | | |
| Severe asthma symptoms (ISAAC) | 20 (-2, 41) | 0.071 | 6488 | 2 (-30, 34) | 0.912 | 6338 |
| Prescribed inhaled corticosteroids | 8 (-30, 46) | 0.686 | 6484 | 8 (-29, 44) | 0.683 | 6334 |
| Asthma admission since age 5 years | -37 (-63, -11) | 0.005 | 6486 | -48 (-68, -28) | <0.001 | 6336 |
|  | Daily number of hours sedentary | | | | | |
| Severe asthma symptoms (ISAAC) | -0.19 (-0.34, -0.04) | 0.016 | 6488 | -0.15 (-0.28, -0.02) | 0.024 | 6338 |
| Prescribed inhaled corticosteroids | -0.20 (-0.30, -0.10) | <0.001 | 6484 | -0.16 (-0.34, 0.02) | 0.076 | 6334 |
| Asthma admission since age 5 years | 0.05 (-0.40, 0.50) | 0.822 | 6486 | 0.02 (-0.20, 0.24) | 0.854 | 6336 |
|  | Daily total steps | | | | | |
| Severe asthma symptoms (ISAAC) | 330 (-62, 723) | 0.099 | 6488 | 239 (-76, 554) | 0.137 | 6338 |
| Prescribed inhaled corticosteroids | -76 (-688, 536) | 0.807 | 6484 | 112 (-378, 602) | 0.654 | 6334 |
| Asthma admission since age 5 years | -350 (-739, 40) | 0.079 | 6486 | -615 (-1509, 278) | 0.177 | 6336 |

^a^Adjustment made for BMI, sex, socioeconomic status, ethnicity, presence of other children in the household, country of residence and household smoking exposure.
