# Supplementary material for: Physical activity among children with asthma: Cross‐sectional analysis in the UK millennium cohort
Source: Pediatr Pulmonol. 2019 Mar 18;54(7):962–9. doi: 10.1002/ppul.24314 (PMC6617805; doi:10.1002/ppul.24314)
Supplement: Supplementary file 4 — Supporting information [file PPUL-54-962-s004.docx]

**Figure S-1 Directed acyclic graph to determine minimum adjustment set**
